# The Carboxy-Terminal αN Helix of the Archaeal XerA Tyrosine Recombinase Is a Molecular Switch to Control Site-Specific Recombination

**DOI:** 10.1371/journal.pone.0063010

**Published:** 2013-05-07

**Authors:** Marie-Claude Serre, Toufic El Arnaout, Mark A. Brooks, Dominique Durand, Johnny Lisboa, Noureddine Lazar, Bertrand Raynal, Herman van Tilbeurgh, Sophie Quevillon-Cheruel

**Affiliations:** 1 Institut de Génétique et Microbiologie, Université Paris-Sud, Orsay, France; 2 Institut de Biochimie et de Biophysique Moléculaire et Cellulaire, Université Paris-Sud, Orsay, France; 3 CNRS, Orsay, France; 4 Plateforme de Biophysique des Macromolécules et de leurs Interactions, Département de Biologie Structurale et Chimie, Institut Pasteur, Paris, France; Saint Louis University, United States of America

## Abstract

Tyrosine recombinases are conserved in the three kingdoms of life. Here we present the first crystal structure of a full-length archaeal tyrosine recombinase, XerA from *Pyrococcus abyssi*, at 3.0 Å resolution. In the absence of DNA substrate XerA crystallizes as a dimer where each monomer displays a tertiary structure similar to that of DNA-bound Tyr-recombinases. Active sites are assembled in the absence of *dif* except for the catalytic Tyr, which is extruded and located equidistant from each active site within the dimer. Using XerA active site mutants we demonstrate that XerA follows the classical *cis*-cleavage reaction, suggesting rearrangements of the C-terminal domain upon DNA binding.

Surprisingly, XerA C-terminal αN helices dock in *cis* in a groove that, in bacterial tyrosine recombinases, accommodates in *trans* αN helices of neighbour monomers in the Holliday junction intermediates. Deletion of the XerA C-terminal αN helix does not impair cleavage of suicide substrates but prevents recombination catalysis. We propose that the enzymatic cycle of XerA involves the switch of the αN helix from *cis* to *trans* packing, leading to (i) repositioning of the catalytic Tyr in the active site in *cis* and (ii) dimer stabilisation via αN contacts in *trans* between monomers.

## Introduction

Organisms with circular genomes can generate chromosome dimers if an odd number of recombination events occur between sister chromatids. These dimers must be resolved into monomers prior to cell division in order to ensure that each daughter cell will inherit a complete genome. In most bacteria, chromosome dimer resolution is performed by two site-specific recombinases, XerC and XerD [1], [2], whereas in archaea only one such protein, XerA, is thought to play this role [3], [4]. The Xer enzymes belong to the tyrosine recombinase family (hereafter referred to as Tyr-recombinase) which is one of the two large classes of site-specific recombinases. Tyr-recombinases are named after their catalytic residue and promote integration, excision or inversion of defined segments of DNA without requiring energy from ATP hydrolysis or *de novo* synthesis of DNA.

Numerous structural and biochemical studies of bacterial and eukaryotic Tyr-recombinases have provided snapshots of the recombination process and detailed description of catalytic and activity control mechanisms. To catalyze recombination, Tyr-recombinases assemble a synaptic complex composed of two DNA sites and four enzyme monomers associated as a dimer of dimers displaying a pseudo-fourfold symmetry. Recombinases then catalyze a stepwise exchange of strands between the two sites to generate recombinant products. In the first step, two of the four recombinases subunits are activated and cleave one strand of each duplex DNA site using the hydroxyl group of the Tyr catalytic residue as a nucleophile. For each site, this results in formation of a 3′-phosphotyrosine DNA-protein covalent complex and release of a free 5′-hydroxyl DNA end. The free 5′-hydroxyl from one strand then attacks the 3′-phosphotyrosine of the partner site, resulting in the first strand exchange and formation of a Holliday junction intermediate. Isomerisation of the synaptic complex activates the second two recombinases, which catalyse cleavage and exchange of the second strands thus resolving the Holliday junction and release of the recombinant sites [5].

A number of Tyr-recombinase structures have been solved, either alone or in complex with substrate DNA (the PDB accession numbers of the structures discussed in this paper are listed in Table S1). Despite a lack of sequence conservation, Tyr-recombinases have a similar two domain structure. This makes a C-shaped clamp into which DNA can be accommodated. The N-terminal DNA-binding domain is separated from a structurally conserved C-terminal catalytic domain by an unstructured linker of about 15 to 20 residues. The catalytic tyrosine is located in or close to the penultimate αM helix. This helix, as well as the last αN helix, are the most variable elements of the highly conserved C-terminal domains. The active site is shared with other enzyme families, and structural studies revealed that Tyr-recombinases share global structural similarities in their catalytic domain with monomeric type IB topoisomerases [6] as well as dimeric telomere resolvases [7].

Several structures of DNA-bound Cre recombinase [8]–[11], IntIA integron integrase [12] or Flp recombinase [13], [14] revealed that, within the tetramer, monomers link one to each other by non-reciprocal αM (Flp) or αN (Cre, IntIA) helix-swap. In the eukaryotic Tyr-recombinase Flp, swapping of the αM helix carrying the catalytic Tyr leads to *trans* cleavage of the target DNA [13].

Several structures of monomers of full-length Tyr-recombinases or isolated catalytic domains have also been described. So far, XerD was the only full-length Tyr-recombinase structure obtained without DNA [15]. The long αN helix of XerD packs to the core of the catalytic domain. As a result the catalytic Tyr is located close to the active site in a conformation that would preclude DNA cleavage [15]. However, repositioning of helix αN would induce rotation of the Cα–Cβ chemical bond of XerD Tyr279 thus placing its side chain appropriately for in-line attack of the scissile phosphate. Biochemical analysis of C-terminal XerC and XerD mutants demonstrated that the terminal helix is involved in protein-protein contacts essential for activity [16]. Finally, the only Tyr-recombinase *apo*-dimer described in the PDB is that of bacteriophage HP1-Int catalytic domain. Within the dimer, the C-terminal αN helices of each monomer are reciprocally swapped [17].

In contrast to bacteriophage-encoded, bacterial and eukaryotic Tyr-recombinases, little is known about archaeal Tyr-recombinases. The only enzyme biochemically characterised is the integrase from the archaeal virus SSV1 [18]–[20]. The structure of its C-terminal catalytic domain has been described very recently [21], [22] and is consistent with a *trans* cleavage mechanism that was proposed on the basis of biochemical and enzymatic approachs [18].

Here we present the first crystal structure of a full-length archaeal Tyr-recombinase, the XerA recombinase from *Pyrococcus abyssi*. XerA displays the canonical two-domain structure of Tyr-recombinases and assembles as an *apo*-dimer. Small Angle X-ray Scattering (SAXS) and Analytical Ultracentrifugation (AUC) experiments revealed that XerA is monomeric in solution at very low concentrations but forms oligomers at higher concentrations. Surprisingly, within the XerA dimer, the C-terminal αN helices dock in *cis* in the groove that accommodates in *trans* the corresponding helices of Cre, IntIA and HP1-Int [10]–[12], [17]. Furthermore the XerA catalytic Tyr261 is extruded from the catalytic pocket in a position equidistant to both catalytic sites in the dimer raising the possibility that DNA cleavage could occur in *trans*. However, using two series of mutants that target (i) active site residues and (ii) the C-terminal αN helix, we demonstrate that XerA follows a *cis*-cleavage mechanism and propose that the *cis*-packing of the C-terminal helix is a key regulator of site-specific recombination.

## Materials and Methods

### Cloning and mutagenesis

The clone constructed in [3] was used to produce an N-terminal His-tagged XerA for enzymatic analyses. R135A and Y261F point mutations were introduced by site-directed mutagenesis using the QuikChange® II Site-Directed Mutagenesis Kit, whereas the ΔαN helix mutant was obtained by PCR. All mutants were His-tagged at the N-terminus. For crystallization a C-terminal His-tagged XerA was produced.

### Purification and crystallization of full-length XerA

The C-terminal His-tagged version of XerA was purified as previously described [3], concentrated up to 3.5 mg.ml^−1^ in 20 mM Tris-HCl pH 7.5, 200 mM NaCl, 5 mM β-mercaptoethanol and immediately crystallized. Diffracting crystals were obtained in 1.8 M ammonium sulfate, 100 mM Hepes pH 7.15, 5% (v/v) ethylene glycol and were transferred into the mother liquor containing 30% ethylene glycol before flash-freezing in liquid nitrogen. X-ray diffraction data were collected on the ID23-1 ESRF beamline. Data processing was done with XDS and XSCALE [23] as well as with the CCP4 suite of programs [24].

### Purification, crystallization and structure solution of the N-terminal domain of XerA

The DNA fragment encoding the N-terminal domain of XerA (residues 1–103) was cloned into pET21. The resulting C-terminal His-tagged recombinant domain was expressed and purified as had been the full length protein. The isolated domain was concentrated to 5.6 mg.ml^−1^ and crystals were obtained from 100 mM Na Citrate, 1.6 M NH_4_SO_4_ and 150 mM K/Na tartrate. Crystals of both unlabeled and Seleno-Methionine-labeled N-terminal domains were cryo-protected using 30% glycerol prior to data collection on the Proxima-1 beamline of synchrotron SOLEIL. Following data processing, while treating Friedel pairs as separate reflections with XDS and XSCALE [23], a search with SHELXD [25] successfully located the anomalous substructure for the two monomers present in the asymmetric unit. These sites were then used to calculate initial phases using SHARP [26]. An initial atomic model was then constructed using BUCANNEER, which was refined using PHENIX.REFINE and finally REFMAC and COOT [27]–[30].

### Structure solution of full-length XerA

The structure of full-length XerA was solved by molecular replacement, firstly by using one chain of the N-terminal domain (residues 7–93) as a search model, using the program PHASER [31]. The N-terminal domain was kept fixed and the C-terminal domain was located using rotation and translation functions, using PHASER. The structure was rebuilt using COOT and refined using PHENIX.REFINE (43,45). Refinement used CNS and finally REFMAC [30], [32]. Residues 1–8, 253–257 and 280–286 are disordered in the crystal.

### Analytical ultracentrifugation

Sedimentation velocity experiments were performed at 25°C using a ProteomeLab XL-I analytical ultracentrifuge (Beckman Coulter) equipped with an AN60-Ti rotor. Detection of the protein concentration as a function of radial position and time was performed by optical density measurements at a wavelength of 290 nm. The protein samples (100 µL at 0.3 mM (11.8 mg.ml^−1^) of XerA in 20 mM Tris-HCl pH 7.5, 1 M NaCl) were loaded in 3 mm thick double sector centrepieces and spun at 42,000 rpm. Sedimentation velocity profiles were monitored at 3 min intervals. Data were analyzed with the software sedfit 12.0 using a continuous c(s) distribution model [33] and Sedphat 8.2 using a self- association model with fitted non-ideality [34]. Hydrodynamic modeling was performed using Hydropro and Us-Somo [35], [36]. The buffer viscosity η and density ρ, calculated using the software Sednterp 1.09, were respectively 0.981 cP and 1.03793 g/ml. All sedimentation coefficients are corrected to standard conditions (20°C, water).

### Small angle X-ray scattering

SAXS experiments were carried out using the Nanostar instrument (Bruker, Karlsruhe, Germany), with X-rays generated by a rotating anode (Cu Ka, wavelength λ = 1.54 Å). The scattered X-rays were collected using a 2D position sensitive detector (Vantec) positioned at 662 mm from the sample. The scattering vector range was 0.011<*q*<0.40 Å^−1^ where *q* = 4πsinθ/λ and 2θ is the scattering angle. Further experiments were carried out at 15°C on the beamline SWING at the synchrotron SOLEIL. The incident beam energy was 12 keV and the sample to detector (Aviex CCD) distance was set to 1927 mm. The scattering vector range was 0.01<*q*<0.50 Å^−1^. Several successive frames (typically 40) of 2 s each were recorded for both sample and pure solvent. We checked that X-rays did not cause irradiation damage by comparing successive frames before calculating the average intensity and experimental errors. SAXS data were averaged and background subtracted using the program package PRIMUS [37]. Intensities were placed on an absolute scale using water scattering. Data were collected in a buffer composed of either 20 mM Tris-HCl pH 7.5, 200 mM NaCl, 5% glycerol or 20 mM Tris-HCl pH 7.5, 1 M NaCl. At low NaCl concentration, the curve was obtained by splicing the data measured at 0.4 mg.ml^−1^ concentration (low-q range) and at 1.8 mg.ml^−1^ concentration (high-q range) in order to avoid the slight effect of oligomerization or attractive interactions observed in the low-q range by increasing protein concentration. The calculated curves were obtained by using the program CRYSOL [38] from the crystal structure by adding missing residues (8 in the N-terminal position and 13 in the C-terminal position including the His_6_-Tag) and using the programs BUNCH [39] and SABBAC [40]. The goodness of fit was characterised by the χ parameter whose square is the average of differences between experimental and calculated intensities (I_exp_(q)−I_calc_(q))^2^ weighted by the experimental errors σ(q)^2^.

### Half recombination sites

Oligonucleotides (purchased from Eurogentec) were 5′ end-labeled by using [γ-^32^P]ATP and polynucleotide kinase. The left half site was labeled on the top strand and the right half site on the bottom strand. Unincorporated nucleotides were removed by spin dialysis. The labeled oligonucleotide was then hybridized with a two-fold excess of unlabeled complementary strand in TE buffer (10 mM Tris, pH 8.0, 1 mM EDTA). When required, the spacer 5′ hydroxyl was phosphorylated by using polynucleotide kinase in the presence of excess unlabeled ATP, prior to hybridization.

### Covalent complex formation

Reactions were carried out in 20 µL of reaction mixture consisting of 30 mM Tris pH 7.5, 50 µg/mL bovine serum albumin, 50 mM NaCl, 25 nM 5′ end-labeled half recombination site and 40 pmol of *P. abyssi* XerA protein. Reactions were incubated for 2 h at 65°C then quenched in Laemmli loading buffer (final concentrations: 40 mM Tris, pH 6.8, 3% SDS, 8% glycerol, 250 mM β-mercaptoethanol, 0.005% bromophenol blue) and heated for 10 min at 100°C. The reaction products were then analyzed by electrophoresis through a 12% SDS-polyacrylamide gel. Products were visualized by phosphorimaging.

### Recombination assays

Half-site recombination assays were performed in the same reaction buffer, and contained a mixture of 25 nM 5′-end labeled half recombination site and 50 nM cold complementary half recombination site. The spacer 5′ hydroxyl end of the labeled site was phosphorylated to prevent self-recombination. After 2 h at 65°C, reactions were stopped by addition of 1 µg of thermolysine and incubation for 1 h at 37°C. Formamide dye mixture (97.5% deionised formamide, 10 mM EDTA, 0.3% bromophenol blue, 0.3% xylene cyanol blue) was added to the reactions and the samples heated for 5 min at 98°C. The reactions were analyzed on a 15% polyacrylamide gel (19∶1) containing 8 M urea in TBE buffer. Electrophoresis was performed for 3 h at 38 V/cm. Product sizes were determined by comparing electrophoretic mobilities of the samples to that of ladders (10 bp DNA step ladder, Promega and 8–32 oligonucleotide sizing ladder, GE Healthcare). Plasmid-based recombination assays were performed as previously described and analysed on 1.2% agarose gels [3].

## Results and Discussion

### Structure determination

**Figure 1:**
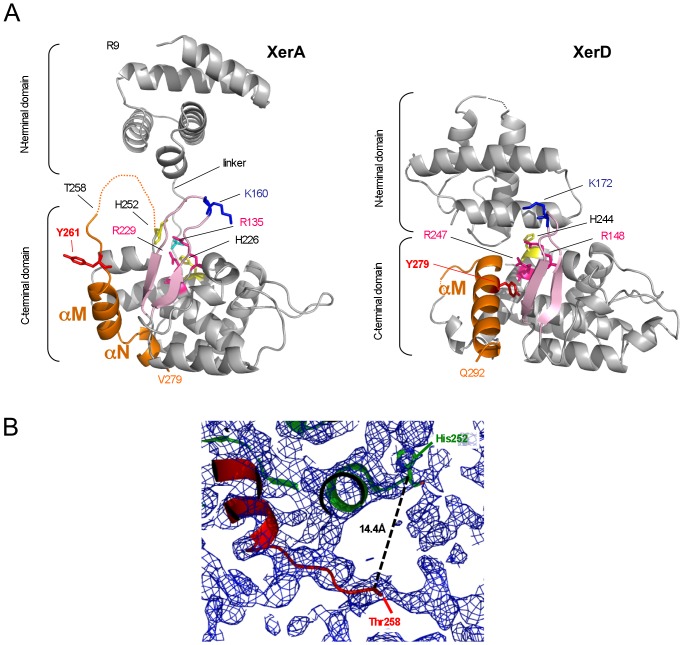
Structure of XerA. **A.** Overall structure of the XerA and XerD *apo*-monomers. Colour code of active site residues: Arg: magenta, Lys: dark blue, His: yellow, Tyr: red. The sulfate ion present in the active site is in cyan. The β2–β3 loop is in pink and the C-terminal αMN helices are orange. The C-terminal His-tag is not visible in the electronic density reflecting its mobility. **C.** 2F_o_-F_c_ electron density map (blue). The backbone of the protein is represented by ribbons (green and red). The distance between the two last visible amino-acids is indicated.

Although diffraction-quality crystals of native XerA were obtained, they could not be reproduced when using the Seleno-Methionine labeled protein. Therefore the structure of full-length XerA was solved by molecular replacement in two stages, using the separate domains independently as search models. Firstly, we used the N-terminal domain of XerA that we solved separately at 3.0 Å resolution (data not shown) as the search model. Secondly, we used the XerD catalytic domain as the search model to solve the XerA C-terminal domain. The final model has a resolution of 3.0 Å and displays good stereochemistry (Table 1). The electron density between H252 and T258 is weak but sufficiently continuous to perceive the link (represented by a broken line in Figure 1). A composite omit map contoured at 0.5 sigma is presented in Figure 1B, and illustrates that the chain does appear to be continuous in this region, albeit with very weak electron density. A search for structurally related proteins in the PDB (Table S1) using the DALI algorithm [41] revealed that the most closely related structure to the N-terminal domain of XerA (residues 9 to 90) is the core-binding domain of λ-Int [42]. Superimposition of these domains gives an r.m.s.d. of 2.22 Å of Cα atoms, with 90% of residues aligned and 12% sequence identity. The most similar structure to the XerA C-terminal domain (residues 107 to 279) is the catalytic domain of IntIA [12], with an r.m.s.d. of 2.3 Å over 152 aligned residues and 41% sequence identity.

**Table 1:** Diffraction data and refinement statistics.

*Diffraction Data*	
Dataset	ID23-EH1
Wavelength (Å)	0.873
Unit cell parameters (Å)	*a* = 92.67, *b* = 157.29, *c* = 45.55
Space group	C222
Resolution limits (Å)†	30–3.0 (3.18–3.0)
Number of observations measured†	30,049 (4,672)
Number of unique reflections measured†	6,939 (1,061)
Completeness (%)†	98.7 (97.1)
<I/σ(I)>†	15.1 (2.38)
Rsym (%)†,§	7.4 (65.4)
Refinement	
Number of non-hydrogen atoms (Protein/water/other)	2,143/3/36
Resolution range (Å)	30.0–3.0
R/R_free_ (%)	21.1/29.3
R.M.S.D. bonds (Å)/angles (°)	0.0095/1.406
<B> (Å^2^) Protein/water/other	66.8/57.4/115.5
Ramachandran Plot (%) Favored/Outliers	95.8/0

† Values in parentheses refer to the highest resolution shell (3.18–3.0Å). § *R*_sym_ = Σ*_h_*Σ*_i_* |<*I*>*_h_*−*I_h,i_*|/Σ*_h_*Σ*_i_ I_h,i_*, where <*I*>*_h_* is the mean intensity for reflection *I_h_* and *I_h,i_* is the intensity of an individual measurement of reflection *I_h_*.

The *R*_free_ was calculated using 341 (4.9%) of the reflections, which were set aside from the refinement.

### Oligomeric state of XerA

Surprisingly, analysis of crystal packing of XerA revealed that two monomers, related by a crystallographic symmetry axis, form a putative dimer (Figure S1). The interface area (defined as the difference between the solvent-accessible surface areas of isolated and interfacing molecules, divided by two) involved in the XerA dimer is 1278 Å^2^, corresponding to 8.2% of the total solvent-accessible area of each monomer, according to PISA [43]. The two N-terminal domains of the XerA dimer contact each other over a very small surface of 214 Å^2^ in comparison to those of Cre bound to DNA (1066 Å^2^). In contrast, the C-terminal domain contacts involve a larger surface in XerA than in Cre (interface buried surface areas: 1720 and 949 Å^2^ respectively). The interactions in XerA are mainly stabilized by contacts between the two αK helices (Figure S1). The dimer significance score calculated by PISA for the C-terminal domains is very high (1.00) confirming the central role of this region in the quaternary structure, whereas the calculated score for the full-length protein is low (0.081), predicting a weak stability of the dimer.

To determine the oligomeric state of XerA in solution, we performed SAXS and AUC analyses. However, to maintain protein solubility at the high concentrations required by these techniques, experiments were performed in a buffer containing 1 M NaCl instead of the 200 mM NaCl containing buffer used for crystallization. SAXS experiments were performed at protein concentrations ranging from 0.2 to 6.6 mg.ml^−1^. At 0.2 mg.ml^−1^ the solution contained only monomers as indicated by the I(0) value, consistent with the data collected in the 200 mM NaCl buffer (see below). As protein concentration increased the scattering intensity I(q)/C strongly increased in the low q region indicating formation of oligomers more pronounced than was observed with a 200 mM NaCl buffer (see below). Unfortunately, at concentrations higher than 2 mg.ml^−1^ the scattering curves at 1 M NaCl were dominated by the contribution of small amounts of large oligomers. The corresponding signal masked scattering of dimer species. AUC was performed at a protein concentration of 11.8 mg.ml^−1^ and revealed that XerA distributes between three main species with sedimentation coefficient values (S_20,w_) of 2.7±0.2 S, 4.4±0.3 S and 6.2±0.3 S, corresponding respectively to the monomer, the dimer and a higher order species that could be a tetramer (Figure S2). Using the crystallographic coordinates of the monomer and dimer, sedimentation values were predicted using different hydrodynamic modeling programs. The predicted values of 2.7 S and 4.3 S for monomer and dimer are in good agreement with the experimental values indicating that the dimensions and shape of the crystallized XerA are consistent with those observed in solution (Figure S2). Finally, the dimer dissociation constant was estimated to be higher than 100 µM, indicating that the monomer-dimer equilibrium is dynamic at this protein concentration and high salt concentration.

**Figure 2:**
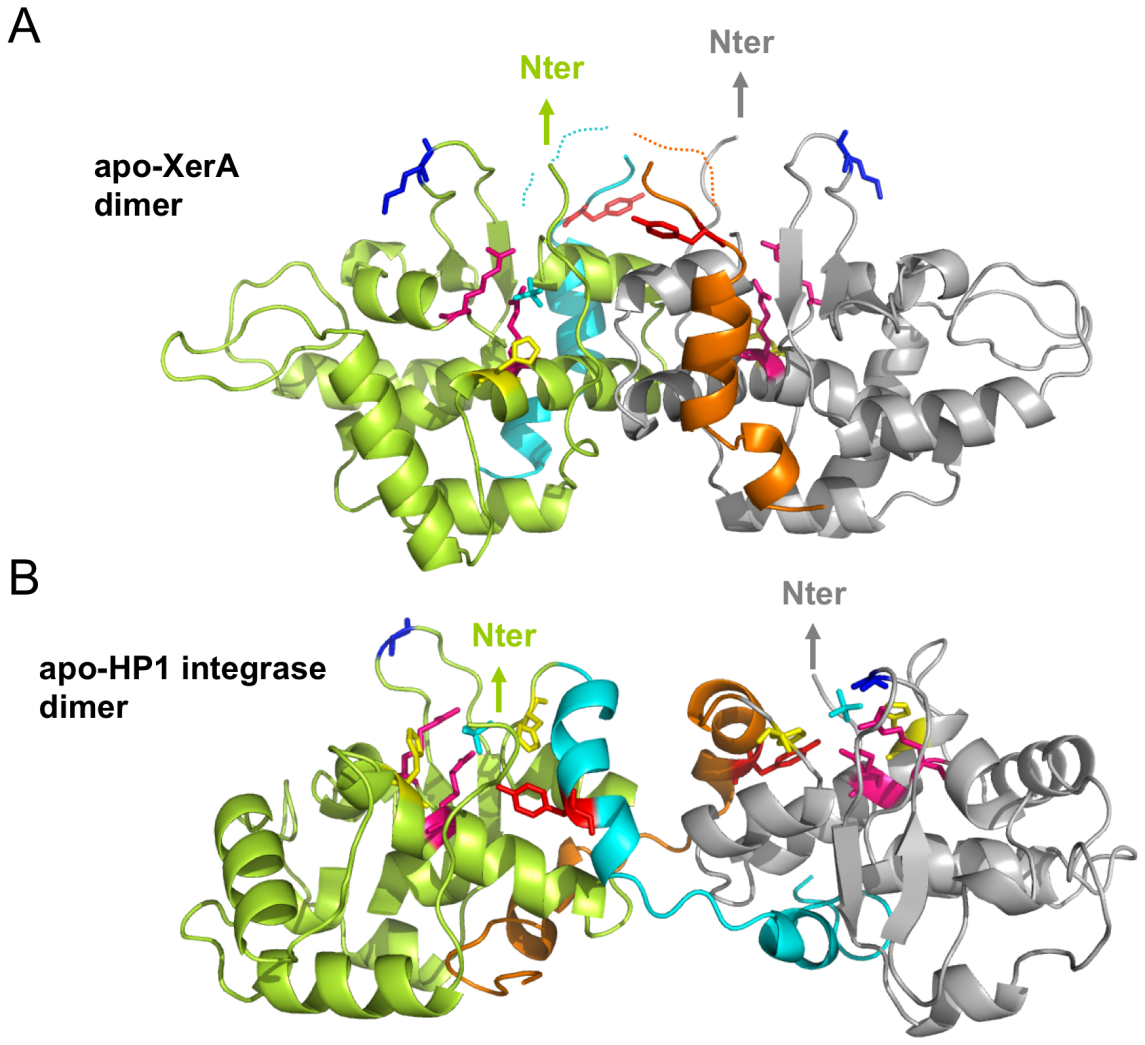
Structure comparison of *apo*-XerA and Cre bound to DNA monomers. *apo*-XerA (grey) and Cre (green) complexed to DNA (gold) are superimposed by their catalytic domains. The XerA catalytic Tyr (Y261) is extruded from the catalytic site. The variable C-terminal αMN helices of XerA are in orange and the C-terminal αMN helices of Cre are in blue. The two N-terminal domains differ in their orientations by about 45°, but exhibit approximately the same concave accessible surface openness.

The XerA dimer differs from DNA bound dimers formed by other Tyr-recombinases. Within the XerA dimer the catalytic domains are related to one another by 180 degrees instead of about 90 degrees in the Cre Holliday junction intermediate (Figure S1). In addition, upon superimposition of the C-terminal domains of XerA and Cre bound to DNA, the N-terminal domain of each XerA monomer deviates by 45° (Figure 2). This deviation suggests that upon DNA binding the XerA N-terminal domain could relocate to clamp DNA, thus repositioning above the C-terminal domain as it is observed for Cre and other Tyr-recombinases (Figure 2).

### The XerA *apo*-monomer displays an open conformation

**Figure 3:**
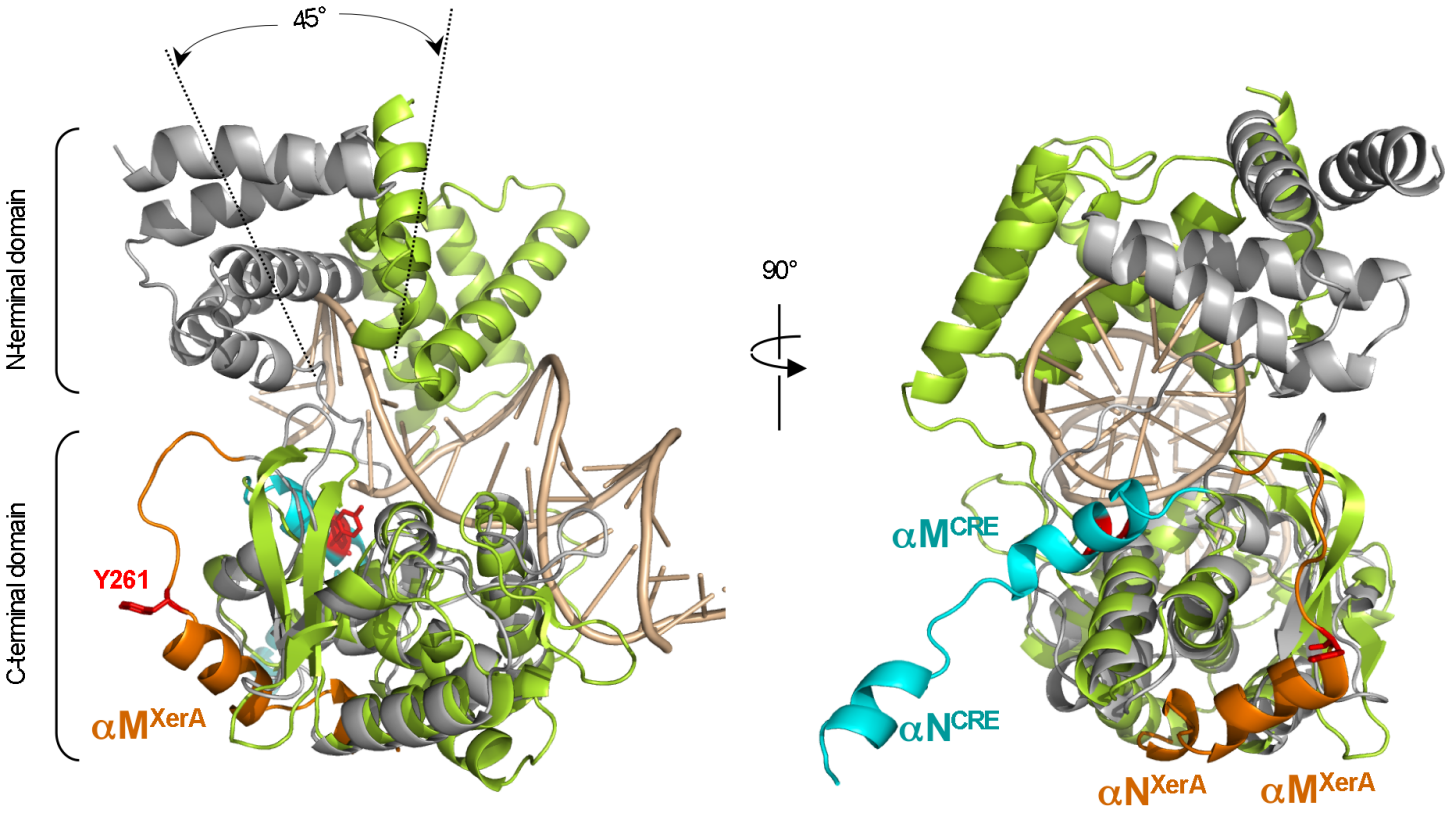
State of XerA in solution at low protein concentration in 200 mM NaCl buffer. **A.** Dots: SAXS experimental data I(q) obtained on the SWING beamline. Red curve: calculated curve from the crystal structure with the missing residues added using BUNCH and SABBAC. **B.** Crystal structure superimposed on a typical envelope of the protein deduced from the SAXS experimental curve using the program GASBOR. The XerA monomer is in grey with the αMN helices in orange. **C.** Four models obtained using the program BUNCH from the crystal structure allowing the N-terminal domain to freely rotate. The grey structure is the crystal structure. All other models are in a variation of pink. For all models the agreement between the calculated curve and the experimental one is excellent (χ≈0.80).

In contrast to the closed structure of the *apo*-XerD monomer from *Escherichia coli* [15], XerA *apo*-monomers present a more open architecture in the crystal. The N- and C-terminal domains are well separated resulting in a marked C-shaped structure for the *apo*-XerA (Figure 1A). This also highlights the conformational freedom afforded by the extended linker. The relative orientation of the XerA N- and C-terminal domains is different from that of the XerD *apo*-structure (Figure 1A). This open conformation is also observed in solution at low concentration as revealed by SAXS experiments (Figure 3). Using the *ab initio* program GASBOR [44], which describes the scattering object as a chain of 292 dummy residues, an envelope of the protein can be deduced from the scattering curve. The typical envelope is extended and accommodates perfectly the crystal structure (Figure 3B). The theoretical scattering curve corresponding to the crystal structure was calculated and corrected for missing residues (8 at the N-terminal and 7 at the C-terminal), then fitted to the experimental curve (Figure 3A). The quality of the fit (χ = 0.80) indicates that the open conformation is present in solution and is not a consequence of crystal packing constraints. Nevertheless the SAXS curve is also compatible with models in which the XerA N-terminal domain possesses some rotational freedom (Figure 3C). Strikingly, the *apo*-XerA open structure is similar to the structures of Tyr-recombinases co-crystallized with DNA (Figure 2) and therefore seems to be in a configuration ready to bind DNA.

### Architecture of *apo*-XerA

The archaeal XerA protein displays the general architecture of Tyr-recombinases with a two-domain structure folded as a C-shaped clamp (Figure 1A). Conservation of the general architecture of Tyr-recombinases from Archaea, Bacteria and Eukarya supports the hypothesis of a common origin for Tyr-recombinases [3]. The N-terminal domain consists of two hairpins, each composed of two anti-parallel helices. These two hairpins are perpendicular to each other in the same cruciform arrangement adopted by the four-helix bundle constituting the core-binding domain of λ Int [42] or the N-terminal domains of XerD [15] and IntIA [12]. Structure comparison of λ Int N-terminal domain in the absence of DNA with the full size λ Int bound to DNA [45], revealed that upon DNA-binding, the N-terminal domain shows only subtle rearrangements, mostly affecting residues involved in DNA contact. The structure of the XerA N-terminal domain may similarly remain unmodified upon DNA binding.

**Figure 4:**
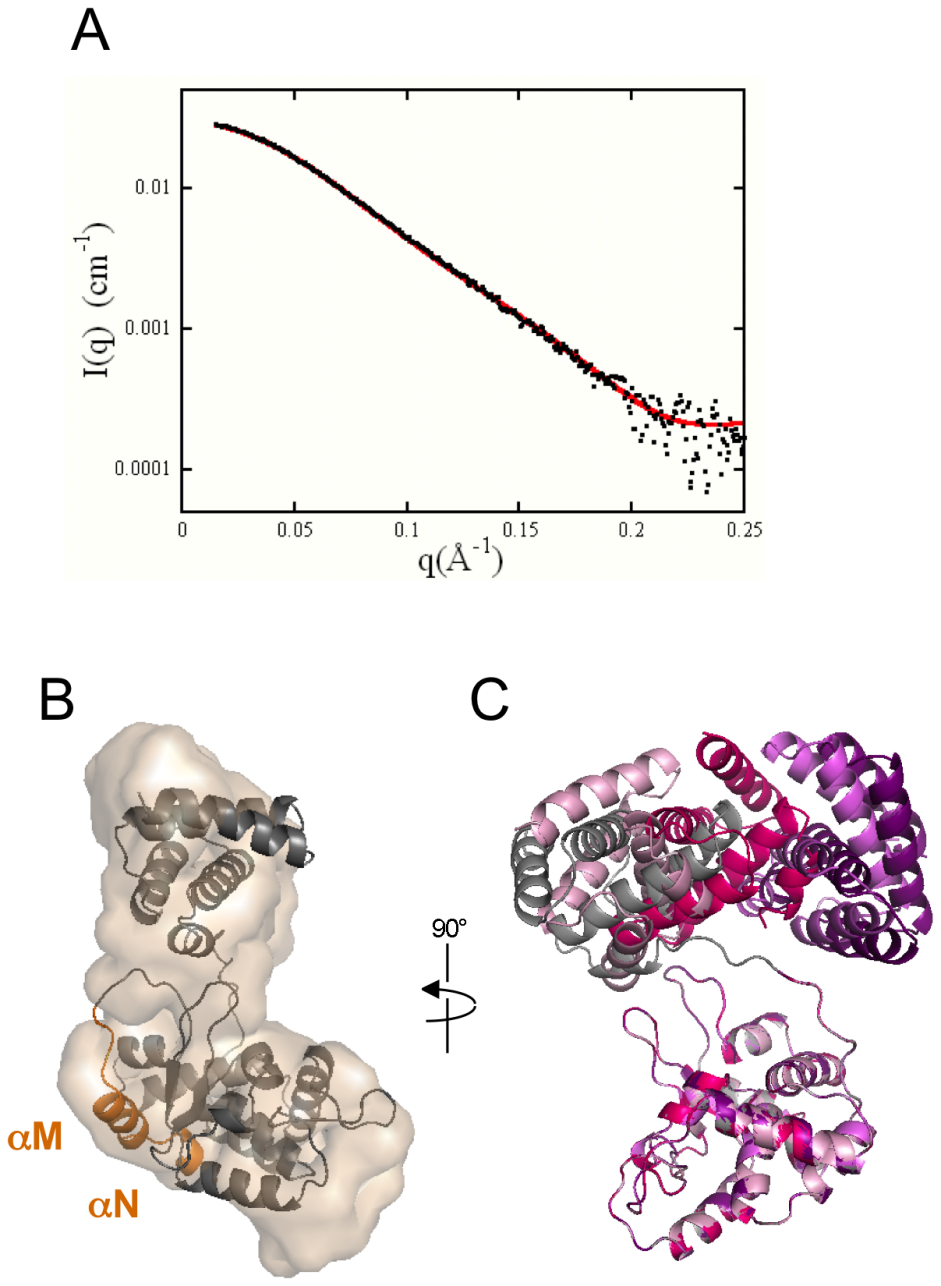
Structure comparison of *apo*-XerA and *apo*-HP1 integrase dimers. C-terminal domain dimers of *apo*-XerA (top) and *apo*-HP1 Integrase (bottom). Within dimers, the C-terminal αMN helices of the green monomer are highlighted in blue and the C-terminal αMN helices of the grey monomer are highlighted in orange. Colour code of active site residues: Arg: magenta, Lys: dark blue, His: yellow, Tyr: red. The sulfate ion present in the active site is in cyan.

The C-terminal domain of XerA is mainly α-helical, except for a 3-stranded β-sheet (Figure 1A). The first short β-strand is not well defined in the structure, as in the case of XerD or HP1-Int. The conserved β2–β3 loop constitutes the edge of the C-shaped clamp of the protein. As observed for XerD, the catalytic residue K160 carried by this loop is removed from the active site (Figure 1A). The β2–β3 loop is fairly mobile and dominates the active site where a sulfate ion is bound both in the XerA and HP1-Int C-terminal domain structures (Figure 4) [17]. The conserved catalytic residues R135 and H226 of XerA are within hydrogen-bonding distance of the sulfate ion. The same coordination is observed in HP1-Int [17]. However, the sulfate ion coordinates the catalytic Tyr in the HP1-Int active site but not in XerA (Figure 4). Interestingly, Y261 is located at the end of the most flexible loop of XerA, at the N-terminus of the αM helix, and is extruded away from the active site with its side chain pointing towards the solvent (Figures 1A and 4). Thus XerA, unlike the HP1-Int catalytic domain [17], or type IB DNA topoisomerase from *Deinococcus radiodurans* [46], only partially preassembles its active site in the absence of the DNA substrate. However, the flexible nature of the loop upstream of the XerA αMN helices may facilitate their repositioning upon assembly of the synaptic complex. The conformational change of αMN helices would be sufficient to direct the catalytic Y261 into the catalytic pocket.

### The αMN helices pack in *cis*

**Figure 5:**
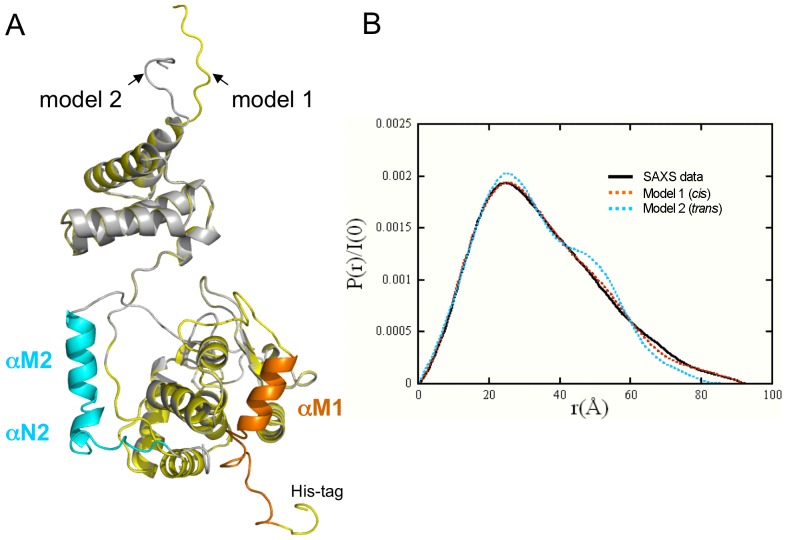
Helices αM and αN pack in *cis*. **A.** Superimposition of the two models constructed from the XerA monomer structure. Model 1 (yellow): *cis* positioning of αMN helices. Model 2 (grey): *trans* positioning of αMN helices. The two possible positions for αMN helices according to each model are in orange and blue respectively. **B.** Distance distribution functions P(r). Black line: experimental curve. Orange dotted curve: XerA monomer with αMN helices packed in *cis.* Blue dotted curve: XerA monomer with αMN helices packed in *trans*.

The weak electron density between residues H252 and T258 in the XerA crystal structure (Figure 1) rendered uncertain the assignment of αMN helices to their parent monomers. SAXS analysis was used to determine the location of the αMN helices in solution. Two models were constructed from the XerA monomer crystal structure (Figure 5A) and addition of the missing residues in the N and C-terminal domains. The first model corresponded to a *cis* positioning of αMN helices and the second model corresponded to *trans* positioning of these helices (*i.e.* helix swapping between monomers). SAXS curves were calculated for both models using CRYSOL [38] and adjusted to the experimental curve. The χ parameters characterizing the goodness of the fit were then compared. The best fit was obtained for the *cis* model (χ value of 0.80) compared to the *trans* model (χ value of 1.25). Moreover, comparison of the distance distribution functions clearly shows that the experimental curve fits better to the curve calculated with the *cis* conformation (Figure 5B). Finally, using SASREF [39] we reproduced the experimental curve by means of a curve calculated from a model based on the crystal structure where the αMN helices are free to move. Strikingly several independent runs starting from either the *cis* conformation or the *trans* conformation converged to similar models where the αMN helices are very close to the *cis* positioning. Therefore SAXS data are compatible with a *cis* positioning of the αMN helices in solution. However, we cannot exclude that it was the compact configuration of the monomer without helix swapping that crystallized.

### XerA can accommodate half site suicide substrates

**Figure 6:**
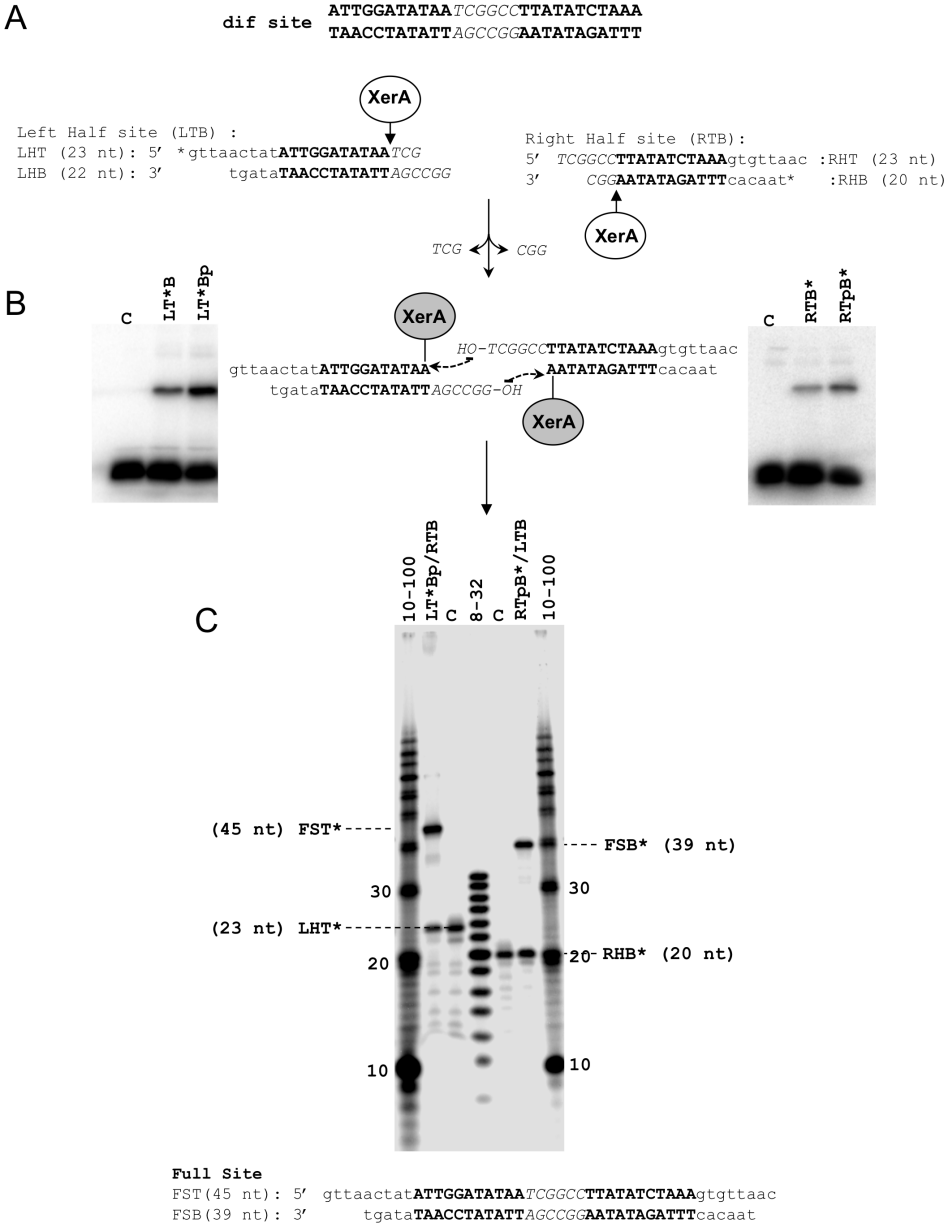
Half-sites strand transfer reactions catalyzed by XerA. **A.** Sequence of the natural *dif* site and half-site substrates. The predicted XerA binding sequence is in bold. The spacer sequence is in italics. The predicted cleavage sites are indicated by arrows. **B.** Covalent complex formation between XerA and half-site substrates was analyzed by 12% SDS-PAGE. Left, reactions on the left half site 5′-end labeled on the top strand. The 5′-end of the bottom strand was either a hydroxyl (LT*B) or a phosphate (LT*Bp). Center, representation of the covalent complexes formed and subsequent steps of the recombination reaction. Right, reactions on the right half site 5′-end labeled on the bottom strand. The 5′-end of the top strand was either a hydroxyl (RTB*) or a phosphate (RTpB*). **C.** Recombination products between half-site substrates were visualized on 15% polyacrylamide-urea gels. The sizes of the top strand exchange (FST*) and bottom strand exchange (FSB*) are indicated and correspond to the predicted product sizes (sequences are presented below the gel).

The reaction catalyzed by Tyr-recombinases involves a transient 3′-phosphotyrosine protein-DNA covalent complex. To identify this complex in XerA, we designed half-site suicide substrates (Figure 6A) similar to those previously designed for λ Int [47] and eukaryotic Flp recombinase [48]. Each half-site contains one of the two XerA binding sites present at the *dif* site and either the 6 nt spacer top strand (right half site) or bottom strand (left half site). Cleavage of these synthetic substrates traps the covalent complex, with the trinucleotide cleaved product diffusing out of the catalytic center (Figure 6A, B). XerA was incubated with either the left or right half-site substrates, and reaction products were analyzed by SDS-PAGE (Figure 6B). The appearance of radiolabeled, low mobility complexes revealed that XerA can cleave and generate 3′-phosphotyrosine covalent complexes with both strands of the *dif* site. This confirmed that the polarity of strand cleavage by XerA is the same as that of other Tyr-recombinases. For both substrates, phosphorylation of the 5′-end of the uncleaved strand resulted in a three-fold increase of covalent complex (Figure 6B). This result strongly suggests that the free 5′ hydroxyl of the uncleaved strand is able to attack the covalent complex either intra- or inter-molecularly as previously observed with the Flp recombinase [48]. Finally, no DNA-protein covalent complex was detected when using the Y261F active site mutant (data not shown), confirming that the catalytic Tyr is required for activity.

To determine the cleavage position within the suicide substrates, recombination reactions were carried out in the presence of both the left and right half-sites with complementary spacer sequences (Figure 6C). The 5′-end of the spacer sequence of the labeled substrate was phosphorylated to prevent illegitimate intra or inter-recombination events. The substrate design is such that product sizes indicate which recombination event has occurred and allows precise localisation of the cleavage position. Analysis of recombination reactions showed that the top strand exchange product FST* is 45 nt long and the bottom strand exchange product FSB* is 39 nt long (Figure 6C). The appearance of both products indicates that strand transfer occurred between left and right half-site substrates and that cleavage positions correspond to the borders of the spacer sequence, a characteristic of Tyr-recombinases [5].

### A *cis* or *trans* cleavage mechanism?

For Tyr-recombinases, one round of recombination requires that four strand-cleavage/strand exchange events are mediated by four recombinase monomers closely assembled within the synaptic complex. Each monomer is bound adjacent to a scissile phosphodiester bond. Two cleavage mechanisms have been described in the Tyr-recombinase family. In *cis* cleavage, each recombinase subunit is catalytically competent and responsible for cleavage of the adjacent phosphodiester bond. In *trans* cleavage, the assembly of the active site requires that the catalytic Tyr be donated from one monomer to the catalytic pocket of another monomer (Figure S3). Hence, the catalytic tyrosine attacks a non-adjacent phosphodiester bond. A *cis* cleavage mechanism is unambiguously indicated by the structures of λ Int, Intl4 and Cre, in the presence of their DNA substrates [11], [12], [45]. Crystal structures of the *apo*-enzymes XerD and HP1-Int also support a *cis* cleavage mechanism [15], [17]. So far only two Tyr-recombinases have been shown to use a *trans* cleavage mechanism, namely the *Saccharomyces cerevisiae* Flp recombinase and the archaeal SSV1 integrase [18], [49]. The crystal structure of Flp revealed that the αM helix, which carries the catalytic Y343 of one monomer, swaps in *trans* to the neighbouring subunit of the dimer of dimers [13]. This results in the swapped Y343 being placed in close vicinity of the active site residues from the adjacent monomer. The recent structure of the Int^SSV^ C-terminal domain shows that the catalytic Tyr is located on a flexible loop that stretches away from the catalytic pocket [21], [22], consistent with the *trans* cleavage mechanism proposed from biochemical data [18].

In XerA the catalytic Y261 is part of a mobile region extending from the end of αL helix to the end of αN helix (Figure 1A). Surprisingly, within the XerA dimer, each catalytic Tyr is almost equally distant from the active sites of both monomers. However, their side chains point toward the second monomer and not toward the active site of their own subunit (Figure 1B). The distance between Y261 and the sulfate ion is of 21.4 Å for a *cis* delivery, whereas it is of 18.24 Å for a *trans* delivery (Figure 1B). Hence, as is the case of the λ Int catalytic domain [50], [51], the structure of the XerA active site does not provide any information on the recombination mechanism, i.e. *cis* or *trans* DNA cleavage. To determine whether XerA acts by a *cis* or a *trans* cleavage mechanism, we constructed two active site mutants, R135A and Y261F. R135 corresponds to the conserved catalytic residue Arg R_I_ [52] and Y261 to the catalytic Tyr. As expected, each mutant alone is inactive in recombination, either on a plasmid or a half-site substrate (Figure S3). *Trans* delivery of the catalytic Tyr to the neighbouring protomer active site would allow partial active site complementation between two different mutants (Figure S3), provided that one mutant lacks the catalytic Tyr and the other modifies one residue of the catalytic pocket [49]. The R135A and Y261F XerA mutants were mixed in equimolar amounts in the recombination assays and activity compared with that of the WT enzyme and mutants alone (Figure S3). No recombinant product could be detected under complementation conditions using either plasmidic or half-site substrates. The simplest interpretation of these results is that XerA follows a *cis* cleavage mechanism despite the unusual orientation of the catalytic Tyr extruded from the XerA dimer. However we cannot exclude that the interactions between the two mutants protomers are different than those between two wild-type protomers. Binding of XerA to the DNA substrate may most likely induce a conformational switch that would close the N-terminal domains around DNA and relocate the loop between αL and αMN helices, directing the catalytic Tyr to the active site pocket of the same protomer.

### Is the αN helix a molecular switch for XerA recombination?

**Figure 7:**
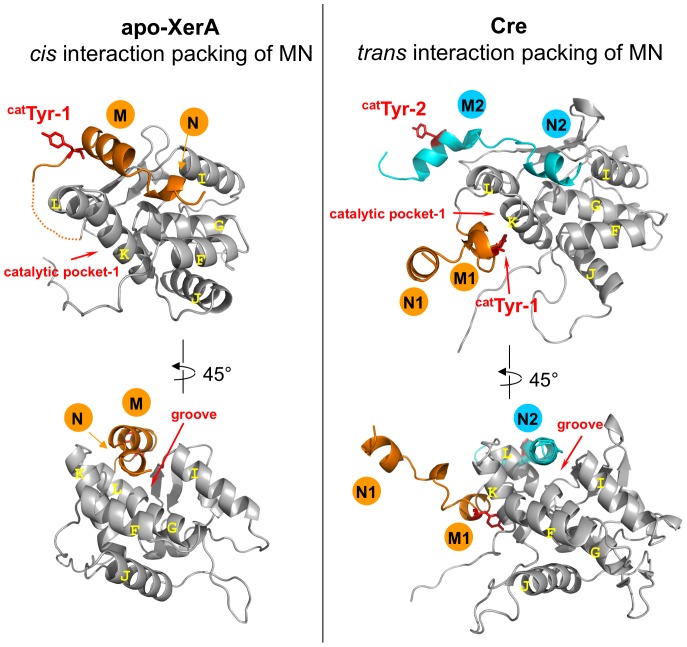
*Cis* or *trans* interactions of αN helices in Tyr-recombinases. The C-terminal domains of XerA and Cre are compared. αMN helices are in orange and catalytic Tyr are in red sticks. For Cre, M2 and N2 helices come from the neighbouring monomer. The groove occupied in *trans* by αN2 helix in Cre is the same as the groove occupied in *cis* by the αN helix of the same subunit in XerA.

The XerA structure revealed that the extreme C-terminal αN helix packs in *cis* into a groove located at the surface of the C-terminal domain (Figures 2 and 7). Comparison with the Cre C-terminal domain structure indicates that the same groove accommodates the αN helix delivered in *trans* from the neighbouring subunit (Figure 7). Notably the contact regions between αN helices and the core domain are similar in *cis* and *trans* packing (Figure 7). Within the Cre dimer bound to DNA, the αN helix of the first molecule (N1) is extruded from the catalytic domain, whereas its αM helix (M1) is oriented toward the catalytic pocket in *cis* (Figure 7). This conformation positions the catalytic Tyr into the catalytic site of the same monomer. Meanwhile, the αN helix of the second subunit (N2) tightly contacts in *trans* the first subunit. The non reciprocal swapping of αN helices contributes to the stabilisation of the dimer. The αN helix is also critical for contacts between XerC and XerD. In fact, interactions between the two recombinases are not critical for the initial steps of recombination but proved to be essential to recombine *dif* sites [16]. Within the XerA *apo*-dimer, positioning of the αN helix in *cis* prevents contacts between the αM helix and the catalytic site resulting in the exclusion of the catalytic Tyr from the active site (Figure 7). It is thus tempting to hypothesize that upon DNA binding, the two last helices (αMN) of the XerA C-terminal domain could relocate in *trans* and induce repositioning of the catalytic Tyr within the active site in *cis*.

**Figure 8:**
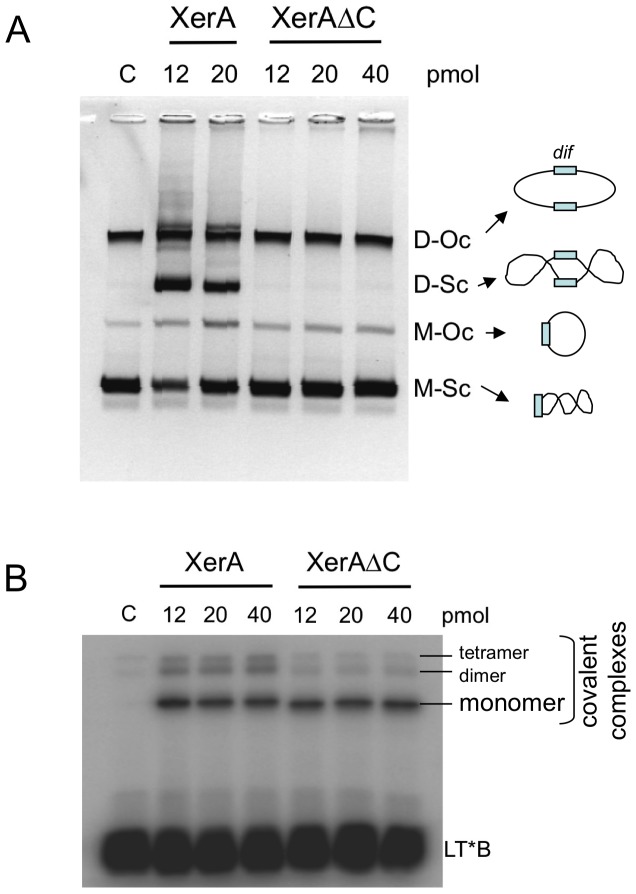
The αN helix of XerA is essential for recombination. **A.** Plasmid-based recombination reactions catalysed by XerA and XerA-ΔC mutant. Recombination products between pBend plasmids harbouring the *dif* site were visualised on 1.2% agarose gels. **B.** Covalent complex formation between XerA or XerA-ΔC mutant and half-site substrates. Reactions on the left half site 5′-end labeled on the top strand. Positions of dimers and tetramers of XerA resistant to thermal denaturation are indicated.

To investigate the role of the αN helix in XerA-mediated recombination, we generated a mutant (XerA-ΔC) deleted for the last 13 amino-acids corresponding to the αN helix alone. XerA-ΔC was expressed and purified following the procedure described for the wild-type enzyme, and its catalytic properties were analyzed. The site-specific recombination activity of XerA-ΔC was evaluated in the *in vitro* assay previously described [3] and compared to the wild-type (Figure 8A). When incubated with a plasmid containing the *dif* site, XerA generates plasmid dimers and multimers to a lesser extent. As protein concentration increases, plasmid dimers are used as substrates and resolved into plasmid monomers. Under the same conditions, XerA-ΔC was unable to recombine the *dif*-containing plasmid even with two-fold excess of protein (Figure 8A). A trivial explanation is that XerA-ΔC is unable to bind and/or cleave the DNA substrate. This hypothesis was tested by using half-site substrates to evaluate covalent complex formation. XerA-ΔC was able to cleave this unconstrained DNA substrate (Figure 8B). However, formation of two additional complexes of high molecular weight, that probably correspond to dimers and tetramers assemblies, was reduced four-fold for the XerA-ΔC mutant as compared to XerA. This observation suggests that deletion of the αN helix severely impairs higher order assemblies required for the recombination process to occur. The interactions between XerA monomers via their C-terminal αN helices are therefore crucial for complete catalytic activity, probably by triggering the catalytic cycle, stabilizing the synaptic complex and the Holliday junction intermediate.

**Figure 9:**
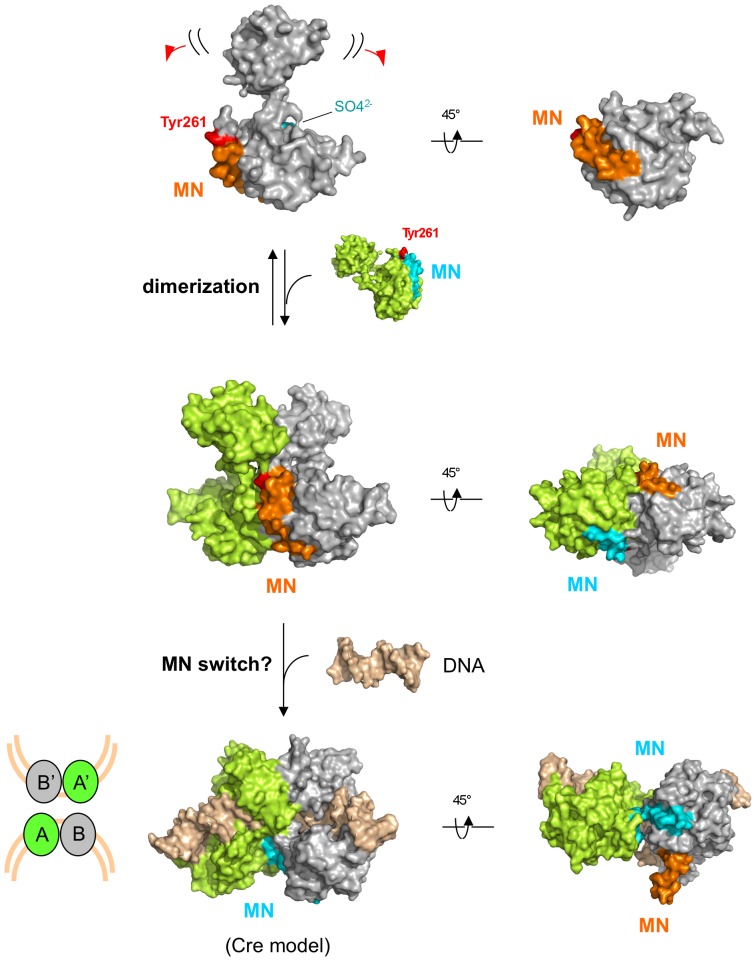
Predicted molecular switches leading to XerA activation. Two monomers of XerA are represented in grey and green. The catalytic Tyr is in red, the sulfate ion in cyan and the αMN helices in orange or blue. The structure of a double stranded DNA is represented in gold. Equilibrium between monomer and dimer conformations is represented by a two-way arrow. The DNA binding step is represented by a single arrow. Two angles of view are represented for each step, showing the switch of αN helices from one subunit to the other. αN helices are not involved in stabilisation of the XerA *apo*-dimer whereas they are essential to stabilise subunits within the synaptic complex. The αN helices switch from the *cis* to the *trans* position induces active site assembly. The last step is illustrated by the X-ray structure of Cre bound to DNA (cartooned at the bottom left with the same colour code). The XerA dimer bound to DNA was inferred from the AB Cre dimer.

The *cis* packing observed for the XerA αMN helices could correspond to a dormant state of the enzyme. Binding to the DNA substrate would induce a switch of these helices towards the catalytic pocket thus initiating the catalytic cycle (Figure 9). Interestingly, the switch would not require dimer dissociation to occur as the XerA dimer interface can accommodate the required αMN helices movement.

## Conclusions

Tyr-recombinases form the largest family of site-specific recombinases, whose members are found in the three kingdoms of life and in the virosphere. In this study we present the first structure of a full-length Tyr-recombinase from the archaeal kingdom, namely XerA from *P. abyssi*. Furthermore, we characterized the enzymatic properties of wild-type and XerA mutants.

The existence of XerA as a monomer at low protein concentration in solution was revealed by SAXS analysis whereas the crystal structure of XerA revealed the existence of a dimer in the absence of DNA. Within this dimer, the two catalytic tyrosines are excluded from the active sites. As observed for the unliganded structure of the λ Int catalytic domain, the catalytic Tyr could in theory be delivered to the catalytic pocket either in *cis* or in *trans* configurations [50], [51]. Enzymatic analyses of XerA active site mutants revealed that the archaeal enzyme follows the *cis* cleavage mechanism. However the C-terminal domain contacts between XerA monomers are different from those previously described in Tyr-recombinases using the *cis* cleavage mechanism (Figure 9). Cre or IntIA assemblies on DNA are stabilized by non-reciprocal swap of their C-terminal αN helices [11], [12] whereas the HP1-Int dimer is stabilised by a reciprocal swap of this last helix [17]. Mutant analyses revealed that the final 5 to 10 residues of XerD are important for contacts with its partner XerC [16], suggesting that the αN helix of bacterial Xer is also involved in the architecture of the Holliday junction intermediate. In contrast, the XerA *apo*-dimer is stabilized mostly by contacts between αK helices of the two subunits (Figure S1), while the αMN helices pack in *cis* in a groove located at the surface of the C-terminal domain. Strikingly, in Cre, IntIA and HP1-Int the corresponding groove is contacted by *trans*-delivered αN helices [11], [12], [17]. As a result of *cis* packing, catalytic tyrosines are extruded from the XerA active site and point towards the solvant. Active site assembly would therefore require remodelling of the dimer, either upon DNA binding or upon tetramer assembly in the synaptic complex. Finally, deletion of XerA αN helix impaired recombination and affected the stability of higher order protein assemblies.

It is therefore attractive to propose that the XerA *apo*-dimer represents a dormant catalytic form in the absence of the recombination site *dif*. The αMN helices could be the molecular switch between dormant and active forms where (i) the active site will be completed by repositioning of the catalytic Tyr and (ii) the tetramer will be stabilised through C-terminal protein-protein interactions (Figure 9).

Whether this particular dimer assembly is a specificity of archaeal enzymes, thermophilic enzymes or a new mechanism to control activity of homodimeric Xer systems remains an open question. Further crystal structures of Xer proteins from different origins will be necessary to elucidate this point.

## Supporting Information

Figure S1 **Residues involved in the XerA dimer interface.** The 2 monomers of both XerA and Cre are in green and grey respectively. **A.** Three 90° rotation views of the XerA dimer. The interaction surfaces are respectively in dark green and black for the green and grey monomers. A close-up of the C-terminal interaction surface is presented in two orientations and residues involved in hydrogen bonds that stabilise the XerA dimer identified by PISA are in sticks. **B.** View of the Cre recombinase in complex with *loxP*. **C.** Close up of the last four helices of XerA C-terminal domain. Two 90° rotation views of the last four helices of the XerA dimer show that contacts occur between helices L and K. Helices M and N are not involved in the dimer interface and pack in *cis* on their respective monomers.
(TIF)

Figure S2 **Sedimentation velocity analysis of XerA at 25 °C.** Detection of the protein concentration as a function of radial position and time was performed by optical density measurements at a wavelength of 290 nm. Main figure: Continuous sedimentation coefficient distribution analysis (inset) Sedimentation characteristic of the monomer, dimer and tetramer forms of XerA calculated with a self association model.
(TIF)

Figure S3 **Organisation of the XerA active site.** **A.** The structure of the active sites within the XerA dimer is at the top, with catalytic residue side chains in orange. One of the two catalytic Tyr is in red. *cis* and *trans* active site organisations are cartooned for wild type and mutants used in complementation assays. Proficient active sites are indicated by a smiley. Only a shared active site model (*trans* delivery of the catalytic tyrosine) restores one of the two active sites present in a dimer. **B.** *Trans*-complementation assay. The recombination efficiency of WT and XerA mutants was tested on a plasmid substrate carrying the *dif* site (left panel) or on the 5′-end labeled left half- site (right panel). Substrates and products for each assay are cartooned. Msc: supercoiled monomer; Moc, open circular monomer. The amounts of protein used in the assays are as follows. C, no protein; WT, 10 pmols; R135A, 20 pmols; Y261F, 20 pmols; YF+RA, 20 pmols each mutant.
(TIF)

Table S1 **List of protein structures discussed in the article.**
(DOC)
