# Correction: The Carboxy-Terminal αN Helix of the Archaeal XerA Tyrosine Recombinase Is a Molecular Switch to Control Site-Specific Recombination

**DOI:** 10.1371/annotation/ad556340-dea0-40c9-b90c-43198d427095

**Published:** 2014-01-02

**Authors:** Marie-Claude Serre, Toufic El Arnaout, Mark A. Brooks, Dominique Durand, Johnny Lisboa, Noureddine Lazar, Bertrand Raynal, Herman van Tilbeurgh, Sophie Quevillon-Cheruel

Multiple Figures were incorrectly switched. The image currently appearing as Figure 2 belongs with the title and legend for Figure 4, the image currently appearing as Figure 3 belongs with the title and legend for Figure 3, and the image currently appearing as Figure 4 belongs with the title and legend for Figure 3. The titles and legends are in correct order. 

