# Supplementary figures and images for: The Carboxy-Terminal αN Helix of the Archaeal XerA Tyrosine Recombinase Is a Molecular Switch to Control Site-Specific Recombination
Source: PLoS One. 2013 May 7;8(5):e63010. doi: 10.1371/journal.pone.0063010 (PMC3646895; doi:10.1371/journal.pone.0063010)

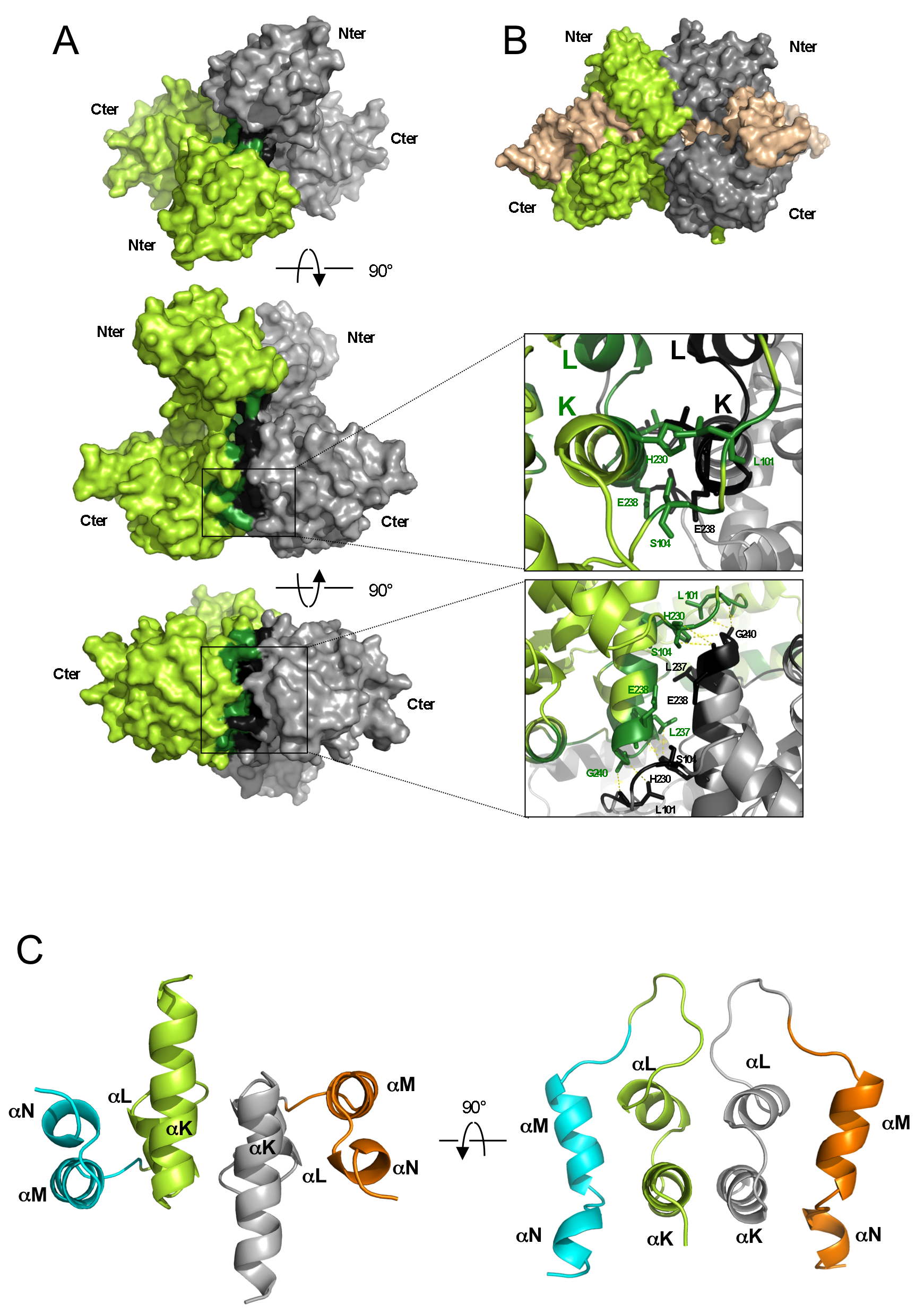

Supplement: Figure S1 — Residues involved in the XerA dimer interface. The 2 monomers of both XerA and Cre are in green and grey respectively. A. Three 90° rotation views of the XerA dimer. The interaction surfaces are respectively in dark green and black for the green and grey monomers. A close-up of the C-terminal interaction surface is presented in two orientations and residues involved in hydrogen bonds that stabilise the XerA dimer identified by PISA are in sticks. B. View of the Cre recombinase in complex with loxP. C. Close up of the last four helices of XerA C-terminal domain. Two 90° rotation views of the last four helices of the XerA dimer show that contacts occur between helices L and K. Helices M and N are not involved in the dimer interface and pack in cis on their respective monomers. (TIF) [file pone.0063010.s001.tif]

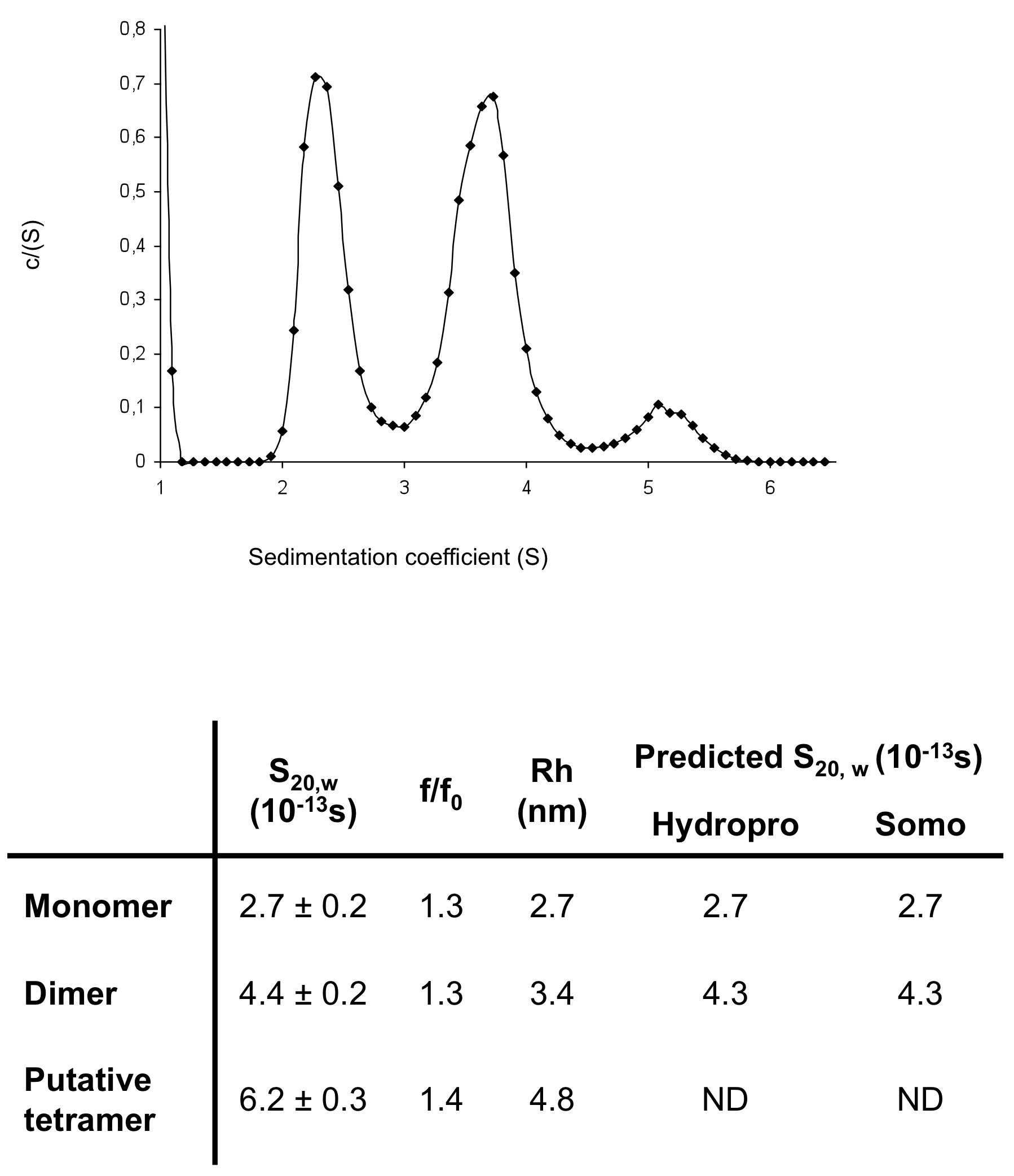

Supplement: Figure S2 — Sedimentation velocity analysis of XerA at 25 °C. Detection of the protein concentration as a function of radial position and time was performed by optical density measurements at a wavelength of 290 nm. Main figure: Continuous sedimentation coefficient distribution analysis (inset) Sedimentation characteristic of the monomer, dimer and tetramer forms of XerA calculated with a self association model. (TIF) [file pone.0063010.s002.tif]

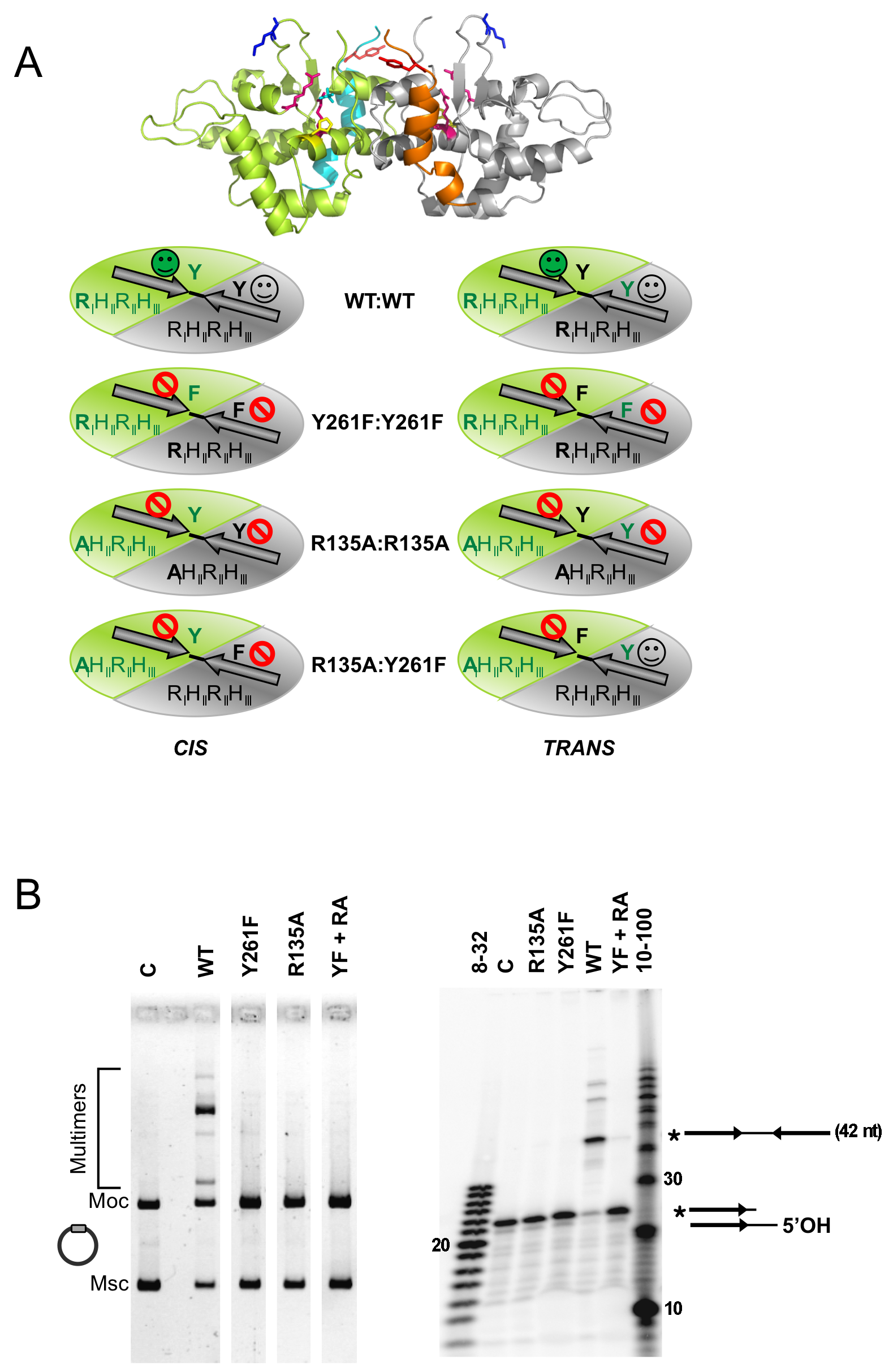

Supplement: Figure S3 — Organisation of the XerA active site. A. The structure of the active sites within the XerA dimer is at the top, with catalytic residue side chains in orange. One of the two catalytic Tyr is in red. cis and trans active site organisations are cartooned for wild type and mutants used in complementation assays. Proficient active sites are indicated by a smiley. Only a shared active site model (trans delivery of the catalytic tyrosine) restores one of the two active sites present in a dimer. B. Trans-complementation assay. The recombination efficiency of WT and XerA mutants was tested on a plasmid substrate carrying the dif site (left panel) or on the 5′-end labeled left half- site (right panel). Substrates and products for each assay are cartooned. Msc: supercoiled monomer; Moc, open circular monomer. The amounts of protein used in the assays are as follows. C, no protein; WT, 10 pmols; R135A, 20 pmols; Y261F, 20 pmols; YF+RA, 20 pmols each mutant. (TIF) [file pone.0063010.s003.tif]
