# Supplementary material for: The Carboxy-Terminal αN Helix of the Archaeal XerA Tyrosine Recombinase Is a Molecular Switch to Control Site-Specific Recombination
Source: PLoS One. 2013 May 7;8(5):e63010. doi: 10.1371/journal.pone.0063010 (PMC3646895; doi:10.1371/journal.pone.0063010)
Supplement: Table S1 — List of protein structures discussed in the article. (DOC) [file pone.0063010.s004.doc]

**Table S1: List of protein structures discussed in the article**

Protein oligomeric DNA site PDB Reference^§^

state accession code

Cre recombinase tetramer *loxP* 3CRX [10]

(Holliday junction intermediate)

Cre recombinase tetramer *loxP* 1CRX [11]

(recombination synapse)

Integron integrase tetramer *attC* 2A3V [12]

(recombination synapse)

Flp recombinase tetramer *FRT* 1FLO [13]

(Holliday junction complex)

bacteriophage λ integrase monomer *attP C’ site* 1P7D [45]

bacterial site-specific recombinase XerD monomer *apo* 1A0P [15]

bacterial type IB DNA topoisomerase monomer *apo* 2F4Q [46]

archaeal site-specific recombinase XerD dimer *apo* 4A8E this work

bacteriophage λ integrase monomer *apo* 2OXO [42]

(core-binding domain)

bacteriophage λ integrase dimer *apo* 1AE9 [51]

(catalytic domain)

bacteriophage HP1 integrase dimer *apo* 1AIH [17]

(catalytic domain)

*§ : References can be found in the article*
